# Supplementary material for: A 2D Convection-Diffusion Model of Anodic Oxidation of Organic Compounds Mediated by Hydroxyl Radicals Using Porous Reactive Electrochemical Membrane
Source: Membranes (Basel). 2020 May 16;10(5):102. doi: 10.3390/membranes10050102 (PMC7280982; doi:10.3390/membranes10050102)
Supplement: Supplementary file 1 [file membranes-10-00102-s001.pdf]

membranes

Supplementary Materials

A 2D Convection-Diffusion Model of Anodic Oxidation of Organic Compounds Mediated by Hydroxyl Radicals Using Porous Reactive Electrochemical Membrane

Ekaterina Skolotneva [1], Clement Trellu [2], Marc Cretin [3] and Semyon Mareev [1,*]

[1] Physical Chemistry Department, Kuban State University, 149 Stavropolskaya str. 350040 Krasnodar, Russia; mareev-semyon@bk.com

[2] Laboratoire Géomatériaux et Environnement (EA 4508), Université Gustave Eiffel, Marne la Vallée, 77454, France; clement.trellu@univ-eiffel.fr

[3] Institut Europeen des Membranes, IEM - UMR 5635, ENSCM, CNRS, Univ Montpellier, 34095 Montpellier, France; marc.cretin@umontpellier.fr

* Correspondence: mareev-semyon@bk.com; Tel.: +7-861-519-9573

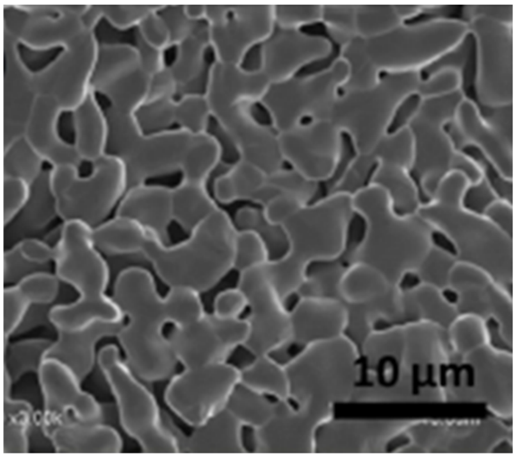

(a)

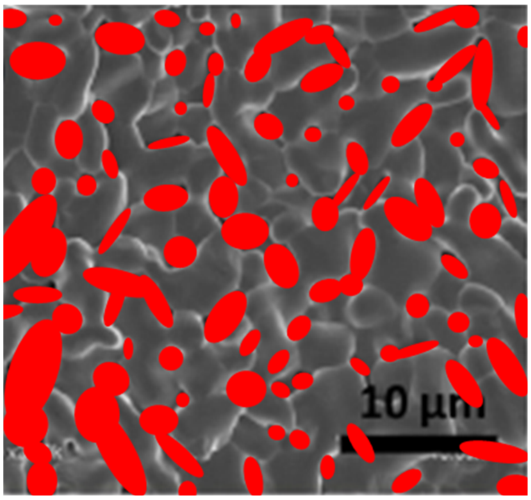

(b)

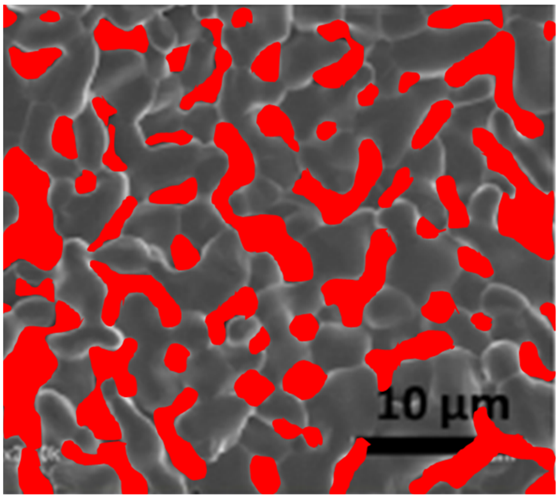

(c)

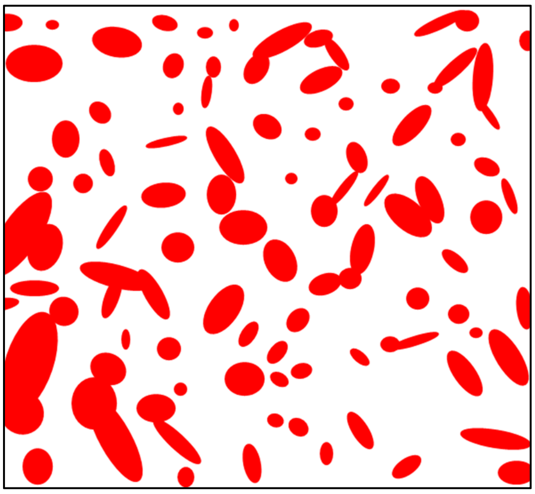

(d)

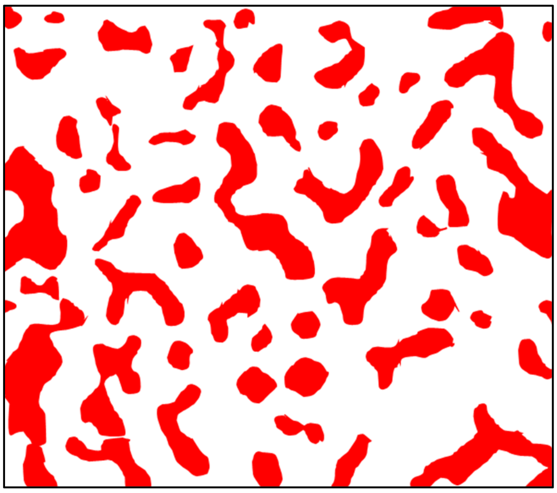

(e)

Figure S1. (**a**) The microphotograph of REM was exposed to a procedure the example of which is presented in the Figure. The pore entrances were painted in red color (in form of oval (**b**) or in random form (**c**)) with the help of Corel PHOTO-PAINT software, and the areas corresponding to electrode material in white color (**d**,**e**). The presence of only a two-color image allowed us to determine the ratio of pores on the REM surface. In both cases (ovals or random form) the relative porous fraction was close to 0.2 (0.195 and 0.21, respectively).
